# Supplementary material for: Accurate graphene quantum Hall arrays for the new International System of Units
Source: Nat Commun. 2022 Nov 14;13:6933. doi: 10.1038/s41467-022-34680-0 (PMC9663594; doi:10.1038/s41467-022-34680-0)
Supplement: Supplementary file 1 — Supplementary Information [file 41467_2022_34680_MOESM1_ESM.pdf]

Supplementary Information for “Accurate graphene quantum Hall arrays for the new International System of Units”

Hans He[1*], Karin Cedergren[1], Naveen Shetty[2], Samuel Lara-Avila[2,3], Sergey Kubatkin[2], Tobias Bergsten[1] and Gunnar Eklund[1].

1: RISE Research Institutes of Sweden, Box 857, S-50115 Borås, Sweden

2: Department of Microtechnology and Nanoscience, Chalmers University of Technology, 412 96 Gothenburg, Sweden

3: National Physical Laboratory, Hampton Road, Teddington TW11 0LW, United Kingdom

*Correspondence and requests for materials should be addressed to Hans He. (email: Hans.he@ri.se)

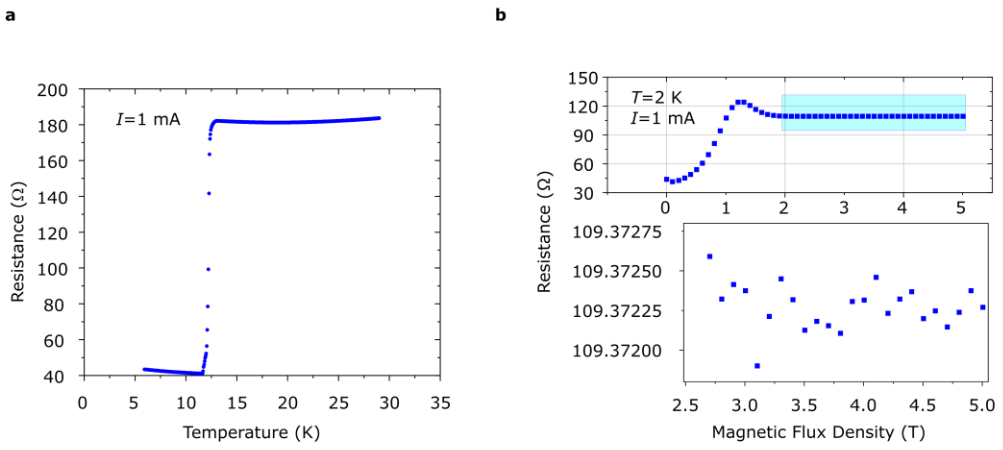

***Supplementary Figure 1. Subarray characterization**. **a,** shows the superconducting transition of the NbN-contacts (critical temperature T_C = 12 K) measured for one subarray. The resistance increase after the superconducting phase transition is due to quantum effects in epigraphene. **b**, shows the magnetotransport characterization of the same subarray, which appears fully quantized after 3 T. This demonstrates that the Hall bar and array have comparable carrier density and mobility. The offset of 4 mΩ (4 μV) from the quantized resistance h/(236e^2), where h is Planck’s constant and e elementary charge, is due to voltmeter error.*

Note that due to its geometry, it is not possible to determine the carrier density or mobility of the subarray via regular Hall measurements. However, since its transition magnetic flux density into quantum Hall effect is comparable to the Hall bar, both above 3 T, their electronic properties must also similar. This is to be expected since the molecular doping method produces homogenous doping, with carrier density differences within 10^{10} cm^{-2} at 2 K.

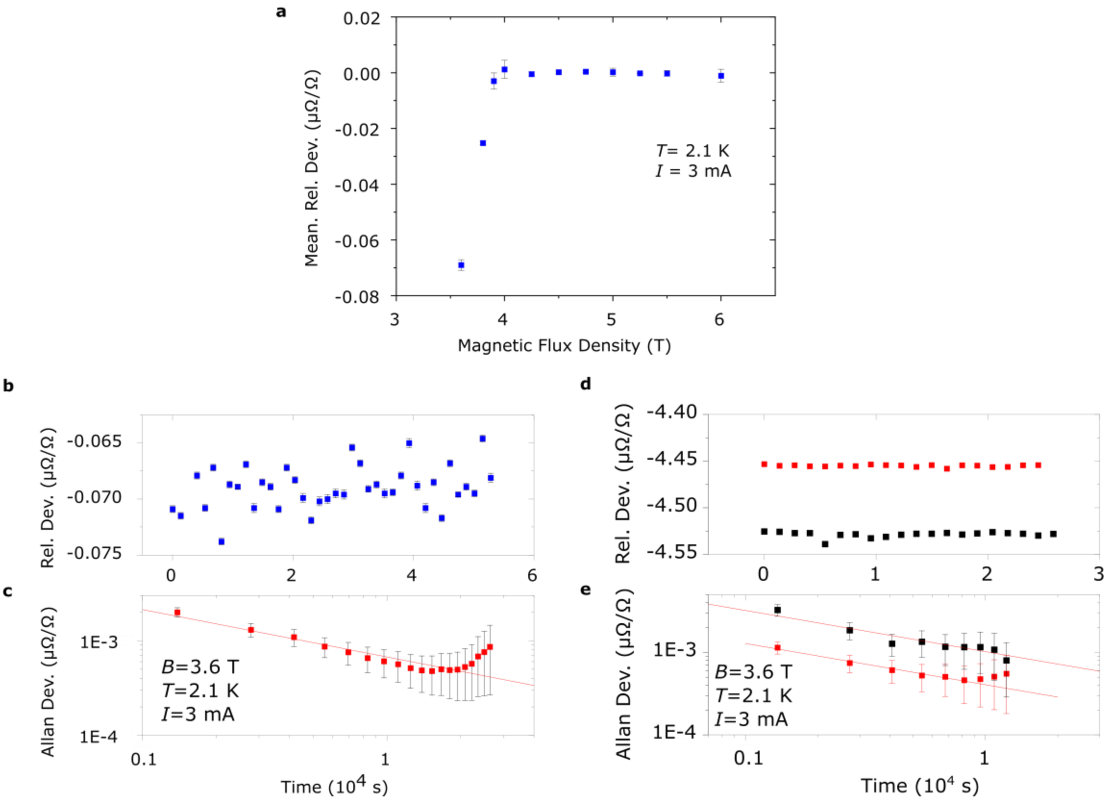

***Supplementary Figure 2. Cryogenic Current Comparator (CCC) comparison measurements between array and Hall bar under non-quantizing conditions. a**, Mean relative deviation versus magnetic flux density for direct comparison of subarrays Array1 vs Array2. The data represent the average of a smaller collection of CCC-readings (20 min each) and the error bars represent one standard deviation (no Allan deviation). Proper quantization is clearly lost below 4 T and there is a significant deviation in resistance. **b** Shows CCC-readings for Array1 vs Array2 in non-quantizing state at 3.6 T. The error bars represent one standard deviation. **c** Shows the corresponding Allan deviation, including estimated error (see Methods). The solid red line shows a fit to $1/\tau^{1/2}$ which is the case for pure white noise, where τ is elapsed measurement time. **d** Shows CCC-readings Array1 (black) and Array2 (red) vs 100 Ω standard. The error bars representing one standard deviation are too small to see. **e** Shows the corresponding Allan deviations, including estimated error (see Methods). The solid red lines show fits to $1/\tau^{1/2}$ which is the case for pure white noise, where τ is elapsed measurement time.*

Supplementary Figure 2a shows cryogenic current comparator (CCC) measurements taken at different magnetic flux densities, and they reveal that the quantization is lost below 4 T. By intentionally measuring the array in non-quantizing state a significant deviation can be produced, and this can be used as an additional test of the comparison measurements.

Supplementary Figure 2b,c shows the direct comparison of Array1 and Array2 at 3.6 T. In this non-quantized state, the subarrays differ significantly, and has relative deviation of $\Delta_{\text{Arra1-Array2}}$ = (-0.0690 ± 0.0006) μΩ/Ω. The uncertainty denotes the standard deviation of the mean, extracted from the corresponding Allan deviation.

Supplementary Figure 2d,e shows a similar comparison of each subarray to a 100 Ω standard. Array1 versus 100 Ω has a relative deviation of $\Delta_{\text{Arra1-100}}$ = (-4.5280 ± 0.0011) μΩ/Ω. The uncertainty denotes the standard deviation of the mean, extracted from the corresponding Allan deviation. Because this subarray is not quantized, the measured value of the 100 Ω standard differs from its nominal value,

which should correspond to a relative deviation of around -4.452 μΩ/Ω as in Supplementary Figure 2a in the main text. On the other hand, Array2 versus 100 Ω has a relative deviation of $\Delta_{\mathrm{Arra2\text{-}100}}$ = (-4.4550 ± 0.0004) μΩ/Ω. The uncertainty denotes the standard deviation of the mean, extracted from the corresponding Allan deviation. This is much closer to the measurement in quantizing conditions. This means that Array1 loses its quantization before Array2, and the indirect comparison has the deviation $\Delta_{\mathrm{Arra1\text{-}Array2,\ indir}}$ = (-0.0730 ± 0.0013) μΩ/Ω, which agrees well with the direct comparison $\Delta_{\mathrm{Arra1\text{-}Array2}}$ = (-0.0690 ± 0.0006) μΩ/Ω. The reason that Array1 and Array2 have different quantizing flux densities is likely due to slight difference in carrier density and mobility. The molecular doping method, while generally homogenous, can yield a finite doping difference on the order of 10^{10} cm^{-2} [28]. Beside the difference in quantizing flux density, once can also reasonably expect a difference in critical current at a given flux density between the two subarrays.

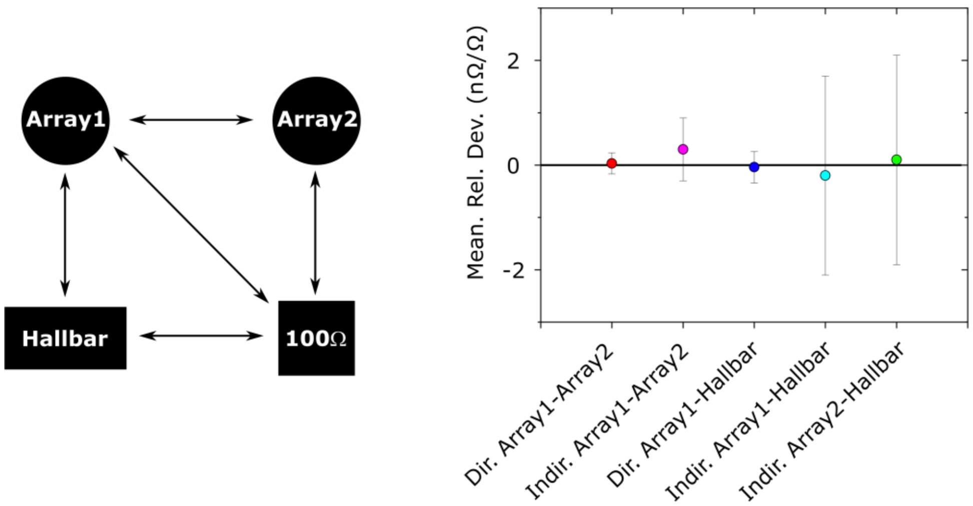

Supplementary Figure 3. Summary of comparison measurements. The arrows in the left diagram depict direct comparisons between different resistance standards. Both direct and indirect comparisons for the subarrays show no significant deviation. The error bars are one standard deviation of the mean, limited by Allan deviation measurements.

In the main test we have demonstrated agreement between different combinations of direct and indirect comparisons between a quantized Hall bar and the subarrays. These measurements are summarized in Supplementary Figure 2, and the weighted mean of all such comparisons is Δ_{Total} = (0.03 ± 0.04) nΩ/Ω, which is zero within the uncertainty. The consistency of the comparison measurements can be checked by looking at the three closed comparison loops. Inside each loop, the relative deviations should sum to zero. For instance, $\Delta_{\mathrm{Array1\text{-}Array2}} + \Delta_{\mathrm{Array2\text{-}100}} + \Delta_{\mathrm{100\text{-}Array1}} = \Delta_{\mathrm{Array1\text{-}Array2}} + \Delta_{\mathrm{Array2\text{-}100}} - \Delta_{\mathrm{Array2\text{-}100}}$ = (0.033 ± 0.62) nΩ/Ω, which is zero within the expanded measurement uncertainty. The other two loops are dominated by the uncertainty of the measurement $\Delta_{\mathrm{HB\text{-}100}}$ and are zero well within an expanded uncertainty of 2 nΩ/Ω.

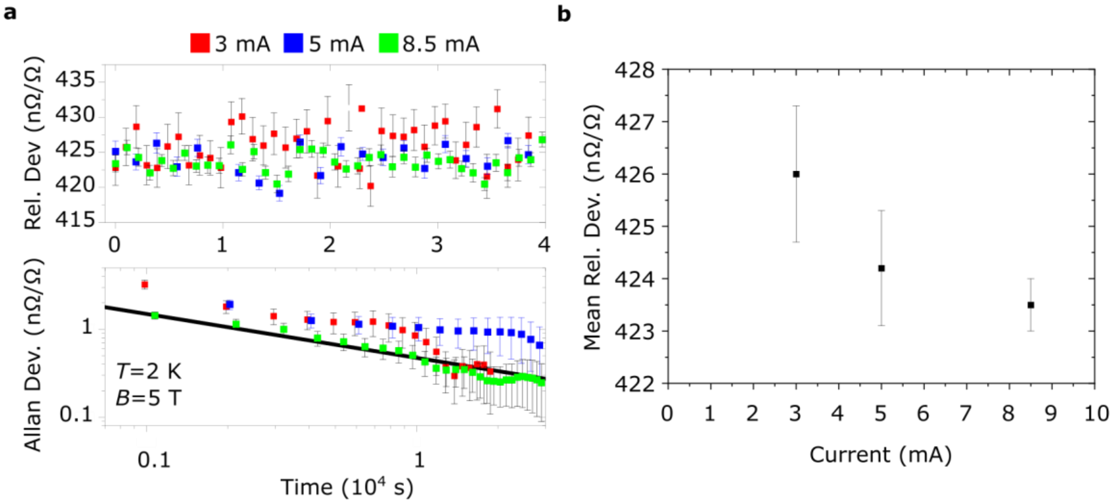

Supplementary Figure 4. Comparison measurements between one subarray and a secondary 12.9 kΩ room temperature standard. a, *Collection of three sets of precision measurements taken at different bias currents. The relative deviation data are given with one standard deviation error bars. The corresponding Allan deviations are shown with estimated error (see Methods). The solid black line shows a fit to* $1/\tau^{1/2}$ *which is the case for pure white noise, where* τ *is elapsed measurement time.* ***b****, The weighted mean of the data in* ***a****, with error bars being one standard deviation of the mean taken directly from Allan deviations in* ***a****.*

In the main test we have shown that at 8.5 mA bias and 5 T there appears to be a slight deviation of around 1 nΩ/Ω between the two subarrays. We wish to demonstrate that this deviation is due to a small deviation from perfect quantization on the order of 1 nΩ/Ω in one of the subarrays, and that it is not the case that they have both deviated very far from quantized conditions in unison. To achieve this, we performed comparison measurements between one subarray and a standard resistor with a nominal value of 12.9 kΩ, kept in a temperature-controlled air bath with a known history. This standard is used instead of the 100 Ω standard described in the main text because it can withstand higher currents. CCC-measurements were performed at different bias currents and the data are summarized in Supplementary Figure 3b. We see that there is no significant change in the relative deviation between bias currents, and it is all within the expanded measurement uncertainty of 3.4 nΩ/Ω (k = 2) for the deviation of 3 mA compared to 5 mA, and 2.4 nΩ/Ω (k = 2) for 5 mA compared to 8.5 mA, and 2.8 nΩ/Ω (k = 2) for 3 mA compared to 8.5 mA. While this measurement is much noisier than the direct subarray versus subarray measurements, it still shows that the quantization of the individual subarray changes at most a few parts-per-billion, limited by noise due to the external 12.9 kΩ standard. For comparison, the precision measurements for the direct comparison between subarray versus subarray, and subarray versus Hall bar, show that the quantization is 0.4 nΩ/Ω (k = 2) for currents 3 mA and 5 mA. This test supports the notion that a deviation on the order of 1 nΩ/Ω develops occurs in one of the two subarrays at high bias currents, similarly to how one subarray loses quantization at lower flux densities before the other. This could be attributed once again to slight differences in electrical properties such as carrier density.
